# Low-Molecular-Weight Heparins: Reduced Size Particulate Systems for Improved Therapeutic Outcomes

**DOI:** 10.3390/molecules23071757

**Published:** 2018-07-18

**Authors:** Fahad Akhtar, Xinyu Wan, Gang Wu, Samuel Kesse, Shaoda Wang, Shuying He

**Affiliations:** 1 School of Life Science and Technology, China Pharmaceutical University, Nanjing 210009, China; tauriansfairy88@gmail.com (F.A.); 15951712208@163.com (X.W.); 18994103736@163.com (G.W.); 18018027031@163.com (S.W.); 2 State Key Laboratory of Natural Medicines, Department of Pharmaceutics, School of Pharmacy, China Pharmaceutical University, 24 Tongjiaxiang, Nanjing 210009, China; kessejnr@yahoo.com

**Keywords:** low molecular weight heparins, stability, encapsulation, polymers, bioavailability

## Abstract

A wide range of diseases have been treated using low-molecular-weight heparins (LMWHs), the drug of choice for anticoagulation. Owing to their better pharmacokinetic features compared to those of unfractionated heparin (uFH), several systems incorporating LMWHs have been investigated to deliver and improve their therapeutic outcomes, especially through development of their micro- and nano-particles. This review article describes current perspectives on the fabrication, characterization, and application of LMWHs-loaded micro- and nano-particles to achieve ameliorated bioavailability. The valuable applications of LMWH will continue to encourage researchers to identify efficient delivery systems that have specific release characteristics and ameliorated bioavailability, overcoming the challenges presented by biological obstructions and the physicochemical properties of LMWHs.

## 1. Introduction

**Figure 1:**
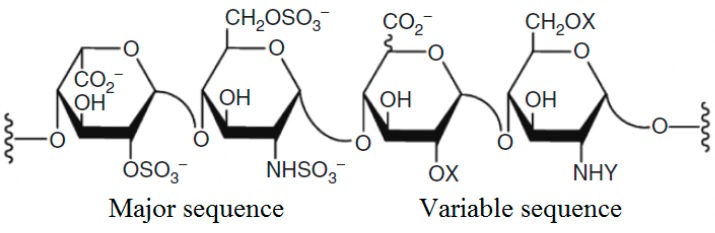
Schematic of the structure of heparin showing its different sequences.

The clinical use of heparin and related compounds as anticoagulants began over half a century ago [1]. Heparin (Figure 1) is a glycosaminoglycan that is structurally similar to heparan sulfate (HS). Which is an endogenous macromolecule that binds to a wide array of protein ligands and is involved in the regulation of a variety of bioactivities, such as blood coagulation, angiogenesis, inflammation, and tumor metastasis [2]. This similarity bestows various features, including anti-inflammatory and growth factor activation, on heparins and low-molecular-weight heparins (LMWHs) [2]. Moreover, different therapeutic activities of heparins [3], such as anti-inflammatory [4] and anticancer [5,6,7,8,9,10,11,12] activities, are also associated with various molecular sequences [13] although absolute specificity is not observed. Indeed, considerable redundancy exists in the system. Major sources of pharmaceutical heparins, including LMSHs, are mammals [14,15,16] in particular, porcine intestinal mucosa. Both heparins, including unfractionated (uFH), and LMWH, are drugs of choice for coagulation disorders, such as thromboprophylaxis and thromboembolism [17,18,19,20,21,22]. 

Encapsulation has played an important role in the synthesis of drug delivery systems, including micro- and nano-particles [23,24,25,26,27]. These modalities are useful for protecting a drug from the environment [28]; controlling the release of a drug [28,29,30,31,32]; and delivering a drug at the desired target site, providing improved therapeutic outcomes [33].

Both uFH and LMWH have different properties, for example, the molecular weight of LMWH is more homogenous, i.e., in the range of 2000–8000 Da, while its anticoagulant response is more predictive and necessitates less coagulation level monitoring than that of uFH [34].

Owing to the biological nature of heparin, its stability in the gastrointestinal environment remains a big issue for formulators, requiring a formulation appropriate for its administration [35]. In fact, the high molecular size, negative charge, hydrophilicity, and poor intestinal permeability of LMWHs have restricted its clinical use to the parenteral route only. For the purpose of improving patient compliance, non- invasive delivery of heparin is required. Various approaches, that include chemical conjugation and encapsulation for the development of non-parenteral systems for the delivery of heparin, have already been documented [36,37]. 

Non-invasive products are not only economical but also offer improved patient compliance [38,39,40]. Various studies have reported the development of micro- and nanoparticles using different polymers, such as alginate, chitosan, and poly-lactic-co-glycolic acid (PLGA) loaded with LMWHs. These novel particulate systems have shown great potential in oral, topical, and nasal delivery of LMWHs, in terms of their effectiveness, safety, and biocompatibility [41,42,43,44].

After an extensive literature search, we could not find a comprehensive review article on particulate systems for delivery of LMWHs, particularly via the different routes. Thus, the objective of this article is to review current perspectives on the development of LMWH-loaded micro- and nanoparticles, particularly for different routes of administration, and to suggest future particulate-based modalities for improved therapeutic outcomes.

## 2. Unfractionated Heparin (uFH) Versus Low-Molecular-Weight Heparins (LMWHs)

Chemical or enzymatic depolymerization of commercial-grade heparin produces low- molecular-weight heparins, which are sulfated oligosaccharides, carry a negative charge, and are hydrophilic in nature [45]. Table 1 describes the procedures for preparing the most frequently utilized commercial LMWHs [46,47]. To exert an anticoagulant effect, LMWHs catalyze Xa inactivation by binding to antithrombin [47]. This results in the suppression of thrombin and inhibition of cascade reactions involving various clotting factors such as fibrinogen and proaccelerin, leading to coagulation [48]. Nevertheless, LMWHs may not be interchanged in clinical use, since they are synthesized by different depolymerization procedures, and thus have different pharmacokinetic and anticoagulation features [49,50]. 

The difference between the pharmacokinetics of uFH and those of LMWHs could be attributed to their reduced availability for interaction with antithrombin, likely due to its higher binding affinity with non-anticoagulent proteins, macrophages, and endothelial cells [48,51,52,53,54]. In addition, uFH-osteoblast binding leads to the increased propensity to cause osteoporosis [55], and an increased tendency of thrombocytopenia is observed when uFH binds with platelet factor-4 [56]. In addition, the anticoagulant effect of uFH is not predictable, probably because of the inconsistency in HBP (heparin binding protein) levels in plasma; thus, vigilant laboratory monitoring during uFH treatment is indicated [52,57]. Conversely, LMWHs exhibit a lower bleeding tendency [58,59], possibly due to a higher proportion of anti-Factor Xa (antithrombotic) than anti-factor IIa (anticoagulant), in which the anti-Xa/anti-IIa ratio is between 2:1 and 4:1, in comparison to uFH, which has a ratio of 1:1 [48]. The clinical benefits of LMWHs over uFH are summarized in Table 2. As a result of these advantages, the clinical use of LMWHs continue to grow. 

**Figure 2:**
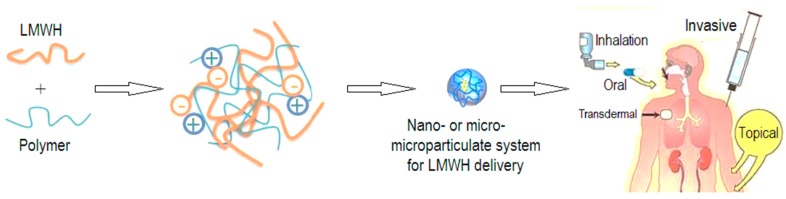
The reduced size particulate systems for delivery of LMWHs through different routes.

Consequently, LMWHs have emerged as a widely accepted anticoagulant of choice for pulmonary embolism and deep vein thrombus [59] and thromboprophylaxis in different diseases [60,61,62], including cancer [5,6,7,8,9,10,11,12,62,63]. LMWHs have been used to treat acute bronchoconstriction [64] and airway hyper-responsiveness [65] in sheep. Furthermore, vessel patency and growth factor activity is maintained during hemodialysis and different vascular diseases, respectively, using LMWHs [66,67]. Moreover, the oral route is not suitable for the delivery of LMWHs, since LMWHs are highly anionic in nature and are thus delivered through the parenteral route only [35,67,68,69,70,71,72]. Conversely, outpatient use of LMWHs is limited owing to its requirement for daily subcutaneous injections [20]. Based on current views, the presentation below summarizes the use of micro- and nano-particles for the delivery of LMWHs through different routes (Figure 2).

## 3. LMWH-Loaded Microparticles

### 3.1. Respiratory Route

Several studies have reported the fabrication and characterization of LMWH-loaded microparticles for different routes. The inhalation of dipalmitoyl phosphatidylcholine microparticles loaded with LMWH achieved therapeutic drug levels and a very swift onset of action, in comparison to subcutaneous treatment [73]. Microparticles in the size range 20–80 µm did not affect drug bioavailability. In addition, LMWH-loaded aerosol microparticles enabled exhibited drug release in the upper respiratory tract and bronchi, without reaching the deep lung. For sustained drug release through prolonged residence time in lungs with improved respirability, large porous PLGA microspheres (diameter 5 µm and density 0.4 g/cm^3^) containing LMWH prepared by a double-emulsion solvent evaporation technique using either stearylamine or polyethyleneamine were capable of enhancing plasma half-life after intra-tracheal instillation of the drug, in comparison with subcutaneous treatment in rats [28]. Therefore, LMWH-loaded, large porous PLGA microspheres were proposed as a practical strategy for sustained drug delivery through the respiratory route. To prove the potential of LMWH against inflammation, polyethylene glycol (PEG) and PLGA-based, large porous microspheres loaded with LMWH were prepared, administered to the sensitized rat animal model of asthma, and observed to have profound anti-asthmatic activity [2].

Several researchers also examined the systemic delivery of LMWHs through the nasal route [74,75]. A potent absorption enhancer, tetradecylmaltoside having non-ionic surfactant properties, was used to formulate nasal drops of LMWH. The results showed an increase in nasal drug absorption, most likely via the time-dependent, reversible opening of tight junctions, and demonstrated that the formulated nasal drops were efficacious and safe [76]. Similar findings were reported when the influence of dimethyl-beta-cyclodextrin on the permeability of LMWH through tight junctions was explored [77]. 

### 3.2. Oral Route

For oral administration of LMWH, various attempts were made to synthesize a microparticulate systems. For drug protection in the harsh conditions of the stomach, acacia gum-gelatin microparticles loaded with Tinzaparin were fabricated through a complex coacervation technique [78]. An in vitro dissolution study showed a biphasic drug release effect: the microparticles efficiently retained the drug at acid pH (less than 4) but allowed swift release of the drug at comparatively basic pH (higher than 4). On the other hand, poor bioavailability of only 4.2% was observed in a rabbit model with only a few Peyer’s patches. This in vivo finding was attributed to the absence of inter- and intra-cellular transport of the microparticles because of their large size (greater than 5 μ m) and diminished likeliness of transport through GALT (gut-associated lymphatic tissue). In a study of the anti-inflammatory effect in inflammatory bowel disease, high entrapment efficiency (up to 78.2%) and pH-dependent release of enoxaparin from Eudragit P4135F microparticles were achieved [79]. A good yield of the Eudragit S100-based pH-sensitive microparticulate system for oral administration of ardeparin was prepared using a spray drying approach [80]. However, the two above-stated studies neither reported the in vitro dissolution nor in vivo properties of Eudragit microparticles. In addition, enoxaparin and nadroparin were loaded to Eudragit RL and RS microparticles using a coacervation technique. The size and the encapsulation efficiency of the prepared microparticles were approximately 40 and 90 μm, respectively; however, the bioavailability was very poor, i.e., <6%. The absorption mode of these LMWHs was attributed to a triphasic process, i.e. first, non-specific adhesion between the microparticles and the mucosal membrane; second, mucin-mediated drug release from microparticles; and, third, diffusion of the free drug across the mucosal membrane [81]. No sign of the polymer effect on the tight junction or microparticle transport across the Peyer’s patch was observed, which might describe the low bioavailabilities achieved. 

Spray dried, colonic microspheres (size ~ 5 µm) of a five component system containing Eudragit L100-55, polyethylene glycol 8000, papain (an absorption enhancer), and LMWH exhibited high encapsulation efficiency (up to 78%), a 6-fold increase in the half-life in comparison with subcutaneous delivery, and targeted delivery of therapeutic quantity of drug at the target site (jejunum) [82]. The ameliorated in vivo findings were attributed to the tight-junction opening role of papain; nevertheless, the bioavailability did not exceed 21%.

### 3.3. Invasive Route

The investigation of invasive delivery of LMWH microparticles is important, because the invasive route is more efficient than the non-invasive. For the prolonged action of enoxaparin, PLGA 85:15 and 50:50 lactide to glycolide, was used to fabricate parenteral microparticles [34,83]. Both studies documented an encapsulation efficiency of more than 50%. PLGA 50:50-based microparticles were also analyzed for their in vitro release and in vivo characteristics. An in vitro dissolution test showed extended drug release effect, while the prolonged drug action was evident from the in vivo study. 

LMWH-loaded multilamellar microvesicles were prepared through a combination of thin film hydration and conjugation techniques. The characterization showed that formulations were not only compatible with blood constituents but also had extended circulatory half-lives [29].

## 4. LMWH-Loaded Nanoparticles

Novel drug delivery systems have been explored globally for the last few decades; however, nanoparticles constitute the most extensively investigated tools, since they can be modified for their size, surface properties, and area to improve their solubility, retention time, and bioavailability [67,84,85,86,87,88,89]. Different sizes of these colloids can be prepared from a large number of polymers, surfactants, and dendrimers. In spite of various issues regarding nano-sized formulations including low encapsulation efficiency, a wide range of size distribution, and scale-up problems (such as failure to maintain particle size and shape), nanoparticles loaded with LMWHs are not only useful for localized drug delivery but also have the potential to penetrating various biomembranes. 

### 4.1. Respiratory Route

In comparing the pharmacological efficacy of ardeparin-loaded conventional liposomes (size ~113 nm) and long-lived pegylated liposomal formulations (size ~104.8 nm) after inhalation in rodent models of pulmonary embolism and deep venous thrombus with that of subcutaneously administered LMWH, similar therapeutic effects were observed after a once daily and a once-every 48 h dosing regimen, respectively [59,90]. Lung tissues did not show any sign of toxicity in any animal. A reduction in half-life and bioavailability were observed in the animals treated with three repeated doses of conventional liposomes, while no such decline was noted in the pegylated treatment. 

Another promising class of nano-carriers is dendrimeric micelles that have been tested for the delivery of LMWHs through the pulmonary route. Approximately 60% and 41% enhancement in the relative bioavailability of enoxaparin from cationic poly(amidoamine) dendrimers with and without pegylation, respectively, was observed, compared with anionic dendrimers that showed no change in bioavailability [70,91]. In addition, a comparable drug efficacy was noted after 100 U/kg and 50 U/kg dose of pegylated dendrimers and subcutaneously administered LMWH, once every 48 h and once every 24 h, respectively.

Nanoparticles containing chitosan (0.11 %), hyaluronic acid (0.17–0.34 mg/mL), and LMWH (0.4 mg/mL) fabricated by an ionotropic gelation technique showed high encapsulation efficiency (approximately 70%) and were able to prevent mast cell degranulation in rats [92]. The nanoparticles exhibited no additional benefit over the pure drug solution in the prevention of histamine release. 

### 4.2. Oral Route

Owing to the efficient uptake of nanoparticles through intestinal lymphatics, there is a continuous increase in the use of nanoparticles for oral drug delivery [88]. The application of nanoparticles in oral delivery of LMWHs is advantageous, since nanoparticles can protect LMWHs against the harsh conditions of the gastrointestinal tract, modify tight-junctions, and undergo adhesion and adsorption to the mucosal membrane. 

To improve the bioavailability of LMWH, its conjugates with lipids were prepared and converted into solid-lipid nanoparticles, which were then stabilized with phosphatidylcholine [93]. The resulting nanoparticles mimicked the chylomicrons, resulting in increased oral bioavailability without gastrointestinal toxicity. This could be attributed to the transcellular transport of solid-lipid nanoparticles through the Peyer’s patches in the GALT.

Chitosan and its derivatives are the most commonly used bioadhesive polymers in preparation of nanoparticles loaded with LMSHs [40,94]. Of these, only one study documented oral bioavailability as 9% [95], while <9% bioavailability was reported by others. In a comparative study of chitosan nanoparticles with and without coating with alginate, coated nanoparticles exhibited 1.6-times higher oral bioavailability than uncoated ones, without affecting the half-life of the drug. 

Hydroxypropylmethyl cellulose phthalate and thiolated chitosan possessing pH-responsive and mucoadhesive features, respectively, were used together to prepare enoxaparin-loaded nanoparticles [71]. This nanoparticulate system exhibited good oral bioavailability of LMWHs (up to 21%).

In addition, chitosan derivatives were used to synthesize poly-electrolyte nanocomplexes of enoxaparin for oral administration. After synthesizing *N*-trimethyl-*o*-carboxymethyl chitosan, carrying a cationic quaternized amino group and a negative charge on the carboxymethyl group, its nanocomplexes with negatively charged enoxaparin were fabricated by a self-assembly technique on the basis of electrostatic interaction [24]. The investigators reported high encapsulation efficiency (up to 81%) and an extended drug release effect for approximately 10 h in the simulated intestinal fluid. However, the Caco-2 cell line treated with nanoparticles revealed an increase in MTT cell toxicity with an increase in therapeutic drug concentration. 

Mucoadhesion properties of glyceryl monostearte were improved up to 18% by grafting them with chitosan [96]. In simulated gastric fluid, only 15% of enoxaparin release was observed after six hours of dissolution. This sustained release behavior was attributed to the strong electrostatic bonding of cationic copolymers with the negatively charged chitosan derivative. In comparison to an oral saline solution of enoxaparin, a single peroral dose of nanocomplexes showed an ameliorated effect on enoxaparin bioavailability (approximately 13%) and pharmacokinetics in the rat model. This could be due to the hydrophobic nature of glyceryl monostearte, the characteristic nature of chitosan, and of the nanoparticles to promote enoxaparin transport across the epithelial barrier of the intestine.

In a different strategy, a polymeric blend comprising Eudragit RS polyester and a positively charged polymethacrylate was utilized to fabricate nanoparticles loaded with tinzaparin [43]. The resulting nanoparticles not only exhibited high oral bioavailability (up to 59%) but also showed an extended anti-coagulant effect (up to 8 h). This successful finding was attributed to the electrostatic interaction between cationic nanoparticles and the anionic mucosal membrane of the intestine. In a similar pattern, bemiparin-loaded nanoparticles were prepared using Eudragit PLGA, RSPO, and a newly synthesized block copolymer from polymethylmethacrylate. High encapsulation efficiency of about up to 98% was achieved. Besides, BaF32 cell proliferation assay in the presence of fibroblast growth factor 2 exhibited an increase in proliferation in a dose-dependent manner, showing the potential of these systems as an enhancer of growth factor function [67]. 

### 4.3. Topical Route

It is notable that various attempts have been made to deliver LMWHs via the topical route for the treatment of superficial thrombosis and hematoma. The topical route was used for the delivery of LMWH-loaded flexosomes (also termed flexible liposomes), carrying different surface charges [44]. The size distribution of flexosomes was 80–170 nm. The positively charges flexosomes exhibited promising features in the context of stability, encapsulation efficiency, and in vitro and in vivo permeation of drugs. Moreover, LMWH-loaded nano-sized liposomes were delivered through the subconjunctival route for the treatment of local hemorrhages [48]. With the aim of minimal systemic side effects, a topical gel was successfully prepared by adding the different LMWHs, nadroparin, bemiparin, enoxaparin, and tinzaparin to a commercially available nanoparticulate suspension fabricated of Eudragit RS [97]. Nanomaterials prepared in different studies were characterized to understand their mode of bioavailability enhancement and fate [41,71].

As described above, taking advantage of the negative charges of LMWHs, the mucosal absorption of LMWHs was improved by fabricating its polyelectrolyte complexes [98]. Conversely, this feature hindered the analysis of LMWHs using common approaches, including capillary electrophoresis, high-performance liquid chromatography, and ion-exchange chromatography coupled with refractive index and ultraviolet-visible detectors [34]. The nephelometric approach [34,79] and azure colorimetric method [91,94] are the most widely used modalities. In addition, a chromogenic assay kit was used to biologically quantify anti-factor Xa [82]. The determination of turbidity produced by a hydrophilic complex between the cationic quaternary ammonium groups of cetylpyridinium chloride and the anionic sulfated groups of LMWH is the principle of nephelometric approach. Azure A is a cationic phenothiazine dye, which is capable of binding with the anionic sulfate groups of the LMWHs. This leads to a decrease in dye absorbance, in a concentration-dependent manner, at 595 nm [70]. In the anti-factor Xa determination method, LMWH is analyzed in the form of a complex with blood antithrombin. The proportional neutralization of FXa to the quantity of LMWH determines the quantity of the LMWH-antithrombin complex. The hydrolysis of a chromogenic substrate by the rest of the FXa amount results in the liberation of a chromophoric group that is tested photometrically.

The valuable uses of LMWHs will continue to motivate researchers to find efficient delivery systems having a characteristic release behavior and improved bioavailability, overcoming the challenges caused by the biological barriers and the physico-chemical features of LMWHs.

## 5. Conclusions

In short, LMWHs are indicated for coagulation, inflammation, and cancer. The anticoagulant effect of LMWHs is more predictable than for uFH. The main problems associated with LMWHs are the relatively short duration of action, the parenteral route of administration, high molecular weight, and their anionic character. Owing to excellent developments in the field of polymer sciences, nanotechnology, and biomedical sciences, the encapsulation of LMWHs has provided promising modes for their effective delivery via micro- and nanoparticulate systems. Through better knowledge of delivery tools and the routes of administration of various LMWHs, LMWH-loaded micro- and nano-particulate systems can be effectively fabricated to overcome LMWH delivery problems, such as protection of LMWHs from the gastrointestinal environment, modulating the release of LMWHs, and delivering LMWHs at the desired target site, providing improved therapeutic outcomes.

## Figures and Tables

**Table 1:** Procedures for the preparation of the most frequently utilized commercial LMWHs from uFH (34).

No.	Procedure	LMWH	Average Molecular Weight	Trade Names
1	Oxidative depolymerisation with hydrogen peroxide	Ardeparin	5500	Normiflo^®^
2	Deaminative cleavage with isoamyl nitrite	Certoparin	5400	Sandoparin^®^
3	Benzylation followed by alkaline hydrolysis	Enoxaparin	4500	Lovenox^®^ and Clexane^®^
4	Oxidative depolymerisation with Cu^2+^ and hydrogen peroxide	Parnaparin	5000	Fluxum^®^
5	Heparinase digestion	Tinzaparin	6500	Innohep^®^ and Logiparin^®^
6	Deaminative cleavage with nitrous acid	Nadroparin	4300	Fraxiparin^®^
7	Nitrous acid deaminative cleavage	Dalteparin	5000	Fragmin^®^
8	Nitrous acid depolymerization, purification through chromatography	Reviparin	4400	Clivarin^®^
9	β-elimination, and fractionation	Bemiparin	3600	Ivor^®^

**Table 2:** Comparative statement regarding the clinical use of uFH and LMWHs.

Features	uFH	LMWH	References
Availability for anti-thrombin reaction	30%	90–100%	[48,51]
Average molecular weight (range)	15 kDa (4000–30,000)	4.5 kDa (2000–10,000)	[26]
Half-life (t_1/2_)	Short (About 1 h (high variability))	Long (3–4 h) (predictable)	[35]
Bioavailability	Low (due to binding with plasma proteins)	Higher than uFH	[35]
Dosage regimen	Frequent dosing (I.V. once/6 h or IV infusion)	Less frequent dosing (IV/SC once/twice daily)	[21]
Clearance mode	Hepatic	Renal largely (thus contraindicated in renal patients)	[48]
Bleeding tendency	High	Lower than uFH	[59]
Thrombocytopenia initiation	High	Lower than uFH	[60]
Osteoporosis propensity	High	Lower than uFH	[13]
Therapeutic response	Variable	Predictable	[34]
Anticoagulant effect	Reversible with protamine sulfate	Limited effect of protamine sulfate	[59]
Laboratory monitoring	Essential	Not required	[59]
